# Quantification of root biomass in barley variety mixtures using variety-specific genetic markers

**DOI:** 10.1186/s13007-025-01464-8

**Published:** 2025-11-12

**Authors:** Mitsuaki Suizu, Björn D. Lindahl, Carsten W. Müller, Thomas Keller, Tino Colombi

**Affiliations:** 1 Department of Soil and Environment, Swedish University of Agricultural Sciences (SLU), Box 7014, Uppsala, 750 07 Sweden; 2 Department of Local Produce and Food Sciences, Faculty of Life and Environmental Sciences, University of Yamanashi, 4-4-37 Takeda, Kofu, 400-8510 Yamanashi Japan; 3 Department of Geosciences and Natural Resource Management, University of Copenhagen, Øster Voldgade 10, Copenhagen, 1350 Denmark; 4 Chair of Soil Science, Institute of Ecology, Technische Universität Berlin, 10587 Berlin, Germany; 5 Department of Agroecology and Environment, Reckenholzstrasse 191, Agroscope, Zürich, CH-8046 Switzerland; 6 School of Biosciences, University of Nottingham, Sutton Bonington, LE12 5RD UK

**Keywords:** Variety mixture, SNP marker, Niche complementarity, Roots, Soil exploration

## Abstract

**Background:**

Variety mixtures combining crop varieties with different root system properties have the potential to improve soil exploration through belowground niche complementarity, which can improve soil resource acquisition and crop productivity. However, there is a lack of appropriate methods to distinguish and quantify roots of different varieties, which limits our ability to elucidate belowground processes that underpin soil exploration and resource uptake by plants in variety mixtures.

**Results:**

In the present study, we developed a method to quantify root biomass and distribution patterns of different barley varieties grown together in mixtures using DNA extraction and quantitative PCR with variety-specific genetic markers. Two field experiments, one in Sweden and one in Denmark, were conducted that included two barley varieties grown either alone in pure stands or together in the same plot. The genetic markers were highly variety-specific, enabling accurate detection of the roots of each individual variety in the mixture. We found that the contribution of varieties to total root biomass in the mixture differed between the two locations, indicating the effects of the environment on root distribution patterns in variety mixtures.

**Conclusions:**

The method presented here opens new possibilities for rapid quantification of root biomass and can provide new insights into belowground processes underpinning the functioning of mixed variety systems. Ultimately, such understanding is needed to assess the potential to adopt mixed variety systems in practical agriculture.

**Supplementary Information:**

The online version contains supplementary material available at 10.1186/s13007-025-01464-8.

## Background

Improving water and nutrient capture of crops from soil is essential to enhance global change resilience of crop production systems [1, 2]. Polycultures, i.e., multiple crop species growing together in the same field, have the potential to improve soil exploration by roots, thereby increasing soil resource acquisition. For example, the polyculture of maize, bean, and squash that combines deeper and shallower root systems allowed complementary exploration of different spatial niches in soil, which may result in yield advantages compared to monocultures [3]. However, the adoption of polycultures is hampered by different biophysical as well as agronomic and socioeconomic constraints. The geographic location and its implications for climatic conditions and the length of the growing season limits the number of crop species that can be grown on a commercial scale at a given location [4]. Furthermore, different crop species can have different sowing and harvest times and require specialised equipment for crop management, which prevents the adoption of polycultures in regions with mechanised agriculture [5].

Variety mixtures combining multiple varieties of the same species with different root system properties in the same field are a potential alternative to polycultures. For example, combining shallow and deep rooting varieties together in the same field can increase the soil volume explored by the crops, thereby increasing resource capture and crop productivity. Compared to polycultures the integration of such variety mixtures into mechanised agriculture is relatively simple. Sowing and harvesting times are consistent between different varieties and field management can be performed with the same equipment [6]. Intraspecific diversity in root traits, such as root growth angle and rooting depth, has been reported for several major crop species including maize [7–10], barley [11], wheat [12], and common bean [13, 14]. Furthermore, a number of studies demonstrated links between root angle and rooting depth, and plant uptake of nutrients and water from different soil layers in maize [8, 15, 16] and wheat [12]. Hence, similar to polycultures [3], mixtures that include crop varieties with different root system properties may foster spatial niche compatibility and thereby improve soil exploration and crop productivity.

A major obstacle towards an improved understanding of the belowground functioning of variety mixtures is the lack of suitable methods that allow rapid variety-specific quantification of root biomass. In the field, root traits are often quantified along excavated trench profiles, with minirhizotrons, or by washing roots from soil cores [17], which all do not allow distinguishing between roots of different varieties. If varieties can be distinguished based on aboveground characteristics, excavated root stocks can be evaluated separately for the different varieties. However, this method is limited to the top 15 to 30 cm of the root system [13, 17, 18] and therefore does not allow elucidation of niche compatibility between shallow and deeper soil layers.

The use of variety-specific genetic markers to quantify root DNA in soil bears potential to overcome these limitations and to quantify root distribution patterns of individual varieties grown together as mixtures. Previous studies successfully quantified species-specific root DNA with quantitative real-time PCR (qPCR), which was then converted to root biomass using regression Eqs [19–22]. This approach can potentially be applied to assess spatial root distribution patterns of different crop varieties in variety mixtures. Genetic markers used to distinguish between species in these studies were located in the internal transcribed spacer (ITS) region [19–21]. However, ITS sequences are not variable enough to distinguish between different varieties of the same species [23]. Therefore, a higher resolution is necessary for variety-specific genetic markers that allow to distinguish roots of different varieties in variety mixtures. Inter-variety genetic variability commonly manifests as single-nucleotide polymorphisms (SNP), describing a single base-pair difference at a specific position in the genome [24]. Thus, a genetic sequence in which a SNP occurs may work as a variety-specific genetic marker to estimate variety-specific root biomass in variety mixtures.

Here, we present a method to quantify variety-specific root biomass distribution patterns in variety mixtures. To do so, we applied qPCR with variety-specific SNP markers. The method was developed using samples from two field experiments with contrasting soil texture in Uppsala, Sweden, and in Taastrup, Denmark. In both experiments, two spring barley varieties (*Hordeum vulgare* L.) were grown either alone (pure stand) or together in the same plot (variety mixture). The abundance of variety-specific SNPs was quantified down to 60 cm soil depth to assess variety-specific root biomass distribution profiles.

## Materials and methods

### Experimental design

Our study was carried out in 2022 in two experimental fields located in Uppsala (59°83′N, 17°71′E), Sweden, and Taastrup (55°40′N, 12°18′E), Denmark, using spring barley (*Hordeum vulgare* L.). Mean temperature and annual precipitation were 7.6 °C and 560 mm in Uppsala (2012–2021) and 9.2 °C and 626 mm in Taastrup (2012–2021). The soil is classified as Cambisol with a silt loam texture (17% clay, 53% silt, 30% sand; uppermost 60 cm) in Uppsala and as Luvisol with a loam texture (18% clay, 31% silt, 51% sand; uppermost 60 cm) in Taastrup. Average soil organic carbon content from the soil surface to 60 cm depth was 1.5% in Uppsala and 0.9% in Taastrup. Dry soil bulk density at 10, 30 and 50 cm depth was 1.36, 1.56, and 1.44 g cm^− 3^, respectively, in Uppsala, and 1.44, 1.46, and 1.44 g cm^− 3^ in Taastrup.

In autumn 2021, both fields were ploughed with a mouldboard plough to ~ 20 cm depth. Harrowing was done before sowing in spring 2022 and fertiliser was applied according to local recommendations for spring barley (Uppsala: 110 kg N ha^− 1^, 5.3 kg P ha^− 1^, 10.1 kg K ha^− 1^; Taastrup: 110 kg N ha^− 1^, 6.9 kg P ha^− 1^, 43.5 kg K ha^− 1^). The field experiments included two spring barley varieties, Feedway and Anneli, which are both grown across Northern Europe. We selected the two varieties based on data from the Swedish national variety trials (2014–2020) to cover a range in plant height (Anneli: 85 cm; Feedway: 62 cm) and grain yield (Anneli: 6.7 t ha^− 1^; Feedway: 8.2 t ha^− 1^). The field experiments included three treatments: Feedway alone (Feedway pure stand), Anneli alone (Anneli pure stand), and a one-to-one Feedway–Anneli mixture (variety mixture). Each plot was 3 m wide and 5 m long. The experiments were arranged as randomised complete block designs with four replications (*n* = 4). The experiments were sown on 13th April 2022 in Taastrup and on 28th April 2022 in Uppsala. The stand density was 350 plants m^− 2^ at a row spacing of 12.5 cm.

### Root sampling

Soil samples for root biomass quantification were collected with a soil auger with 3 cm inner diameter at flowering (BBCH 65) from 20th to 22nd June, 2022 in Taastrup and from 28th to 29th June in Uppsala. The soil samples were collected at five randomly selected points between crop rows within a 0.5 m^2^ subplot located in the centre of each plot. To quantify depth distribution of root biomass, samples were subdivided into three depth increments, i.e. 0–20, 20–40, and 40–60 cm. The five samples taken from each plot were pooled depth-wise and frozen at -20 °C within a few hours after sampling.

### DNA extraction

Frozen samples were homogenised in a custom-made grinder, and 50 mL subsamples were freeze-dried, followed by ball-milling at a frequency of 30 s^− 1^ for 30 s (Mixer Mill MM 400, Vender Scientific, Haan, Germany). Grinding jars and balls were washed with laboratory detergent after every sample to avoid DNA contamination between samples, followed by ethanol rinsing for drying. Root DNA was extracted from approximately 450 mg of soil using the NucleoSpin^®^ Soil Kit (Macherey-Nagel, Düren, Germany) following the manufacturer’s instructions with the following modification: for the homogenisation step, we used a Precellys 24 Touch Homogenizer (Bertin Technilogy, Montigny-le-Bretonneux, France) with two cycles of 5000 rpm for 30 s with a 30 s interval. The extracted DNA was quantified using a Qubit^®^ fluorometer (ThermoFisher Scientific, Waltham, MA, USA). Samples with initial DNA concentrations ranging from 2 to 5 ng/µL were diluted to a standard level, whereas extracts with low DNA concentrations (< 2 ng/µL) were not diluted.

To test for primer specificity and construct plasmid standard curves (see below), we extracted root DNA from Feedway and Anneli roots following the same procedure as described above (i.e. with the NucleoSpin^®^ Soil Kit; Macherey-Nagel, Düren, Germany), using roots from four-week-old plants that were grown in pots. The roots were thoroughly washed and freeze-dried before DNA extraction.

### Design of variety-specific primers

Variety-specific single nucleotide polymorphism (SNP) markers were used to distinguish root DNA of the different varieties. For this, DNA was extracted from seeds (five seeds per variety) and genotyping was done with the iSelect Illumina Infinium 15 K SNP chip by TraitGenetics GmbH (Gatersleben, Germany) as described in [25]. We identified 1417 genetic sequences with a SNP site for Feedway and 653 genetic sequences with a SNP site for Anneli. To distinguish between the two varieties, forward primers were designed with the 3´-end corresponding to the SNP site and evaluated in silico using ThermoFisher´s Multiple Primer Analyzer and Primer3web [26–28]. Primer specificity was tested by PCR amplification in a SimpliAmp Thermal Cycler (ThermoFisher Scientific, Waltham, MA, USA) with 25 µL PCR reactions containing 30 ng of DNA template, 10× Dream Taq Buffer (ThermoFisher Scientific, Waltham, MA, USA), 0.2 mM of dNTPs, 0.025 U/µL of DreamTaq DNA Polymerase (ThermoFisher Scientific, Waltham, MA, USA), and 2 mM of MgCl_2_ as a final concentration. A variety of different primer concentrations and annealing temperatures were evaluated. We selected a set of specific primers targeting a SNP marker in BOPA1_10669-188 for Feedway and in BOPA1_1286 − 990 for Anneli (Table 1 and Supplemental Fig. S1).

**Table 1:** Sequences of feedway and Anneli specific primers, the product size, and the type of nucleotide mismatch at the 3´-end of the forward primers

Variety	Primer	Forward primer sequence	Amplicon length	Mismatch at 3´ end
Feedway	Forward	5´-CCTGGAACCGATCTACCCA-3´	107 bp	A/C
	Reverse	5´-CCTAGGGACATGGAAGCTGT-3´		
Anneli	Forward	5´-GATCACTTCATGGTTTCCCCTC-3´	140 bp	C/C
	Reverse	5´-GACATCACTACGGGAACAAGC-3´		

### Construction of plasmid standards

Feedway and Anneli SNP markers were amplified from root DNA, and the PCR products were cloned with the TOPO TA Cloning^®^ Kit, the pCR^®^4-TOPO^®^ Vector, and One Shot^®^ TOP10 chemically competent *Escherichia coli* (ThermoFisher Scientific, Waltham, MA, USA) following the manufacturer’s instructions. Plasmid DNA was extracted from bacterial cultures with the GeneJET Plasmid Miniprep Kit (ThermoFisher Scientific, Waltham, MA, USA) and quantified using a Qubit 4 fluorometer. After linearization with the SpeI restriction enzyme (New England Biolabs, Ipswich, MA, USA), SNP marker inserts were purified using the E.Z.N.A.^®^ Cycle Pure Kit (Omega Bio-tek, Norcross, GA, USA) and then used to produce qPCR standards.

### Biological standards to convert marker copy numbers to root biomass

To enable conversion of copy numbers of Feedway and Anneli SNP markers to dry root biomass, we established biological standards (separate standards for each combination of variety and soil from the two field locations). For this, we used thoroughly cleaned and freeze-dried roots of four-week-old, pot-grown Feedway and Anneli plants (four plants from separate pots per variety) and soil from spots adjacent to the experimental fields in Uppsala and in Taastrup that were not cultivated with barley in recent years. Using roots from pot-grown plants enabled us (i) to avoid any contamination of the samples used to construct the biological standards with unwanted plant material and (ii) to ensure that finer and coarser roots were included in the biological standards. Soil was mixed with either Anneli or Feedway roots at concentrations of 0, 0.1, 0.5, 1, 2, 2.5, and 5 mg dry root biomass per g dry soil. The root-soil mixtures were ball-milled, and DNA was extracted following the same procedure as described above, with three extracts produced for each mixture as technical replicates.

### Quantitative real-time PCR

Abundances of Feedway and Anneli markers were quantified using quantitative PCR (qPCR) in a CFX384 Real-Time System (Bio-Rad, Hercules, CA, USA). The 20 µL qPCR reactions contained iQ SYBR^®^ Green Supermix (Bio-Rad, Hercules, CA, USA), 0.4 µM (each) of Feedway or Anneli primers, and up to 25 ng of DNA template. The qPCR conditions, optimised for variety specificity, had an initial denaturation step at 95 °C for 3 min, followed by 40 cycles of denaturation at 95 °C for 30 s, annealing at 65.8 °C (for Feedway primers) or 67.2 °C (for Anneli primers) for 30 s, and a final step of 10 s at 80 °C, at which fluorescence was measured. The reactions were finished with a melting curve starting at 55 °C with an increase of 0.5 °C per 5 s up to 95 °C.

Potential inhibition in all extracts and the biological standards was tested by amplifying a DNA fragment including the Anneli marker carried by the pCR^®^4 TOPO^®^ Vector either in sample or in buffer, with M13 primers targeting sites in the vector. Inhibition was not detected in any sample or biological standard. All qPCR plates were prepared with both plasmid and biological standards, as well as non-template controls. All reactions were prepared with three qPCR replicates by an automated robotic 96/384 qPCR setup system (Rob: AlphaHelix, Nacka, Sweden). Samples with deviating melting curves were removed from further analysis.

### Statistics

Statistical analyses and data visualisations were performed using R version 4.3.0 [29]. Linear regressions between estimated marker copy number and root biomass were established based on the biological standards using the ggpmisc R package version 0.5.5 [30]. The depth above which 75% of the estimated root biomass occurred was linearly interpolated for every plot and used as a proxy for root system depth. Analysis of variance was used to test the effects of variety, site, management (pure stand vs. mixed varieties), and their interactions on the depth above which 75% of the estimated root biomass occurred (all factors were set as fixed). Normal distribution of residuals was checked with Shapiro-Wilk tests with statistical significance set to *p* = 0.05. Least significant difference tests were carried out using the agricolae package for R [31]. Data was visualised with the R packages ggplot2 version 3.4.4 [32] and ggpubr version 0.6.0 [33].

## Results

### Location-specificity of the biological standards

**Fig. 1:**
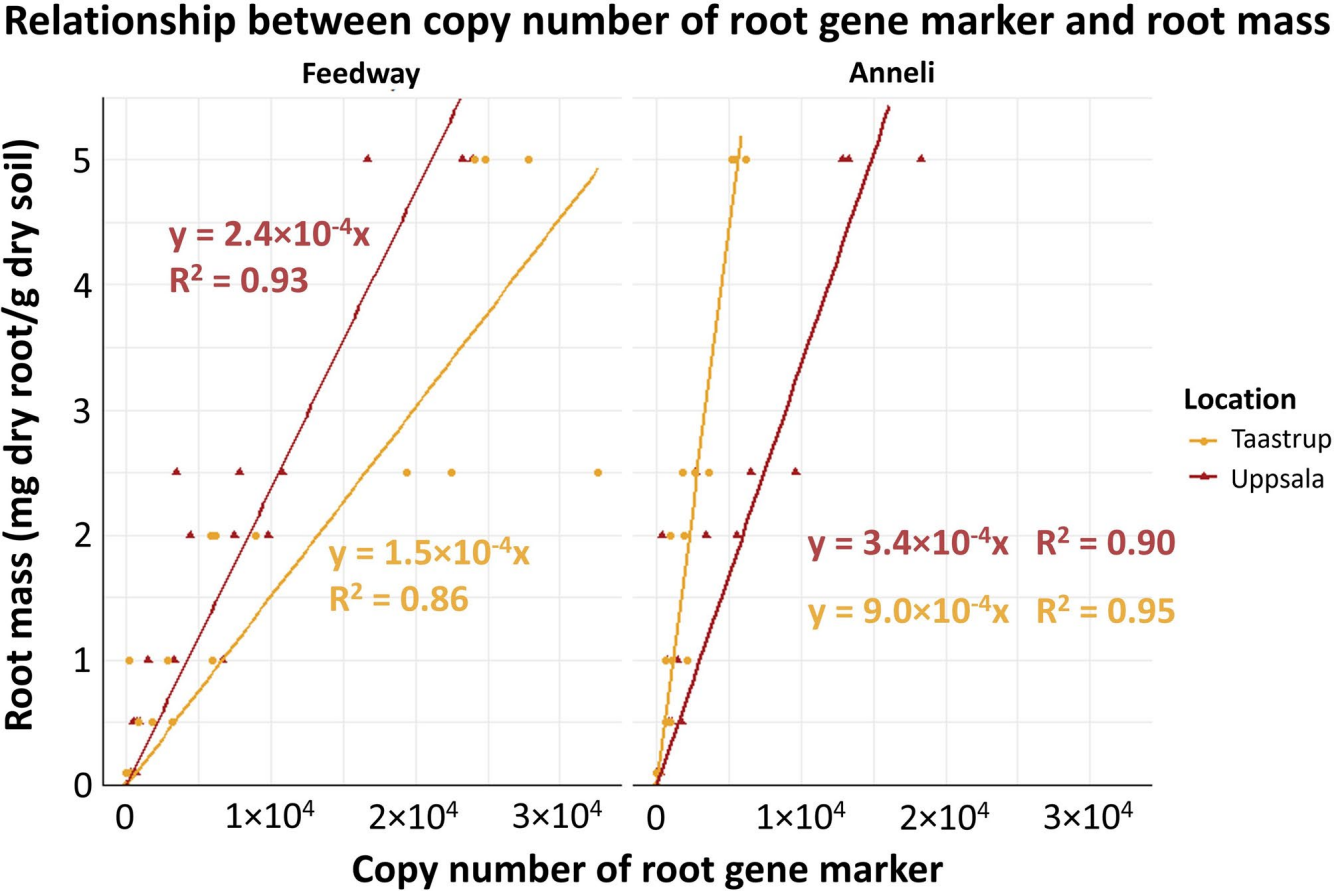
Biological standards used for converting the copy numbers of Feedway and Anneli SNP markers to their root biomass in soil from the field in Uppsala and Taastrup. The intercept of the linear regressions was set to 0. All coefficients of the linear regressions were statistically significant at *p* = 0.001; R^2^ values denote coefficients of determination

Plasmid standard curves of Feedway were linear (R^2^ = 0.989 to 0.999) with an amplification efficiency ranging from 82.6 to 89.5%. Plasmid standard curves of Anneli were also linear (R^2^ = 0.982 to 0.997) and showed a similar amplification efficiency ranging from 83.9 to 92%. In the biological standards, marker copy numbers were significantly correlated (*p* < 0.001) with root biomass (mg root/g soil) in both locations with R^2^ values ranging from 0.86 to 0.95. For Feedway the slope of the regression was considerably steeper in Taastrup than in Uppsala, whereas for Anneli, the slope of the regression was considerably steeper in Uppsala than in Taastrup (Fig. 1).

### Primers showed a high degree of variety specificity

The pure variety stands allowed us to test the specificity of the designed primers (Table 1). In Uppsala as well as Taastrup, the two primer sets were highly specific for their targeted variety. No or negligible amplification occurred when Feedway primers were used on samples containing Anneli roots, and vice versa (Supplemental Fig. S2).

### Root biomass distribution in pure stands

**Fig. 2:**
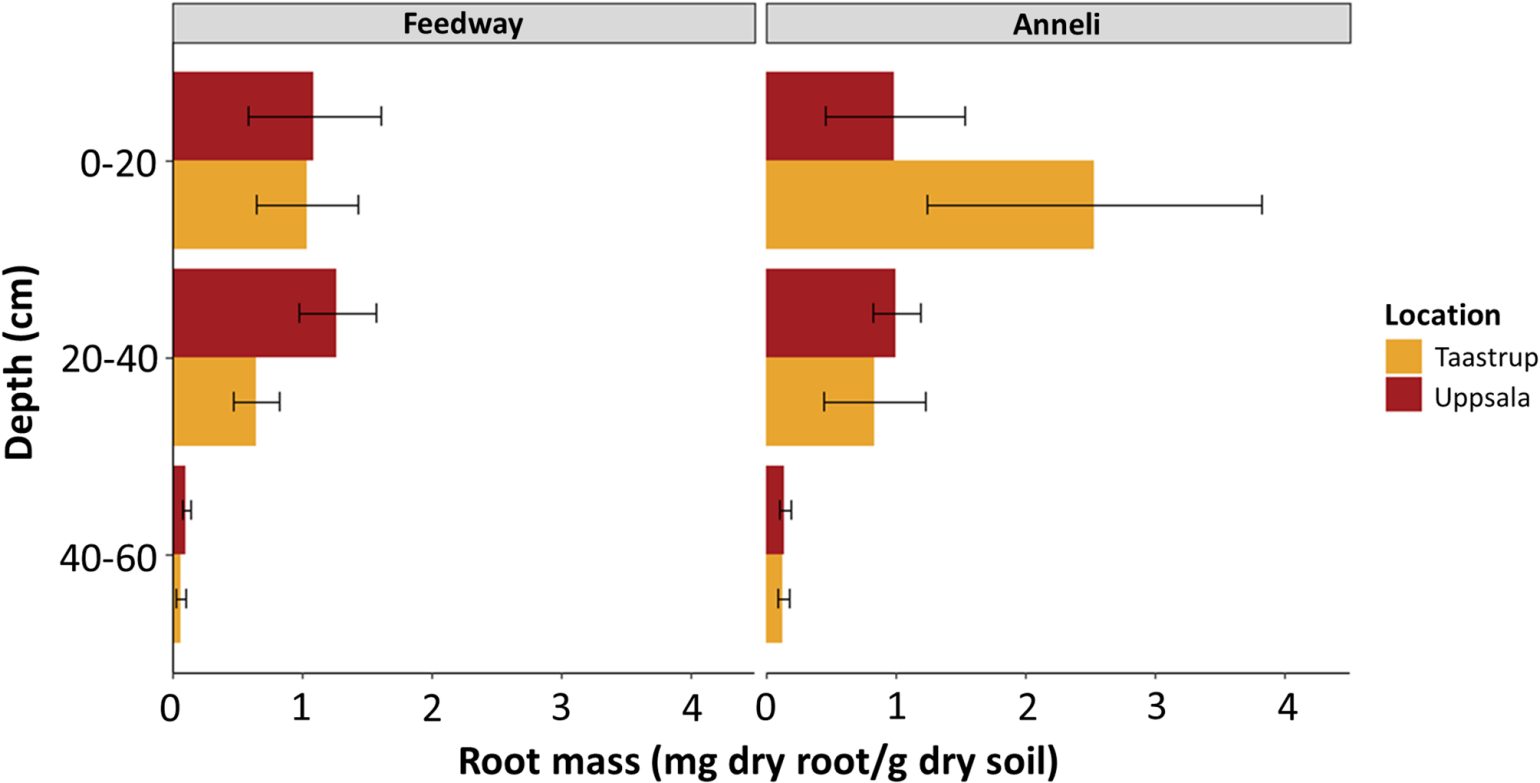
Root biomass of Feedway and Anneli grown in pure stands across different soil layers. The bars represent the mean values of Feedway and Anneli root mass in Uppsala and Taastrup. Error bars represent standard errors (*n* = 4)

In both locations, a general trend of decreasing root biomass with increasing soil depth was observed in pure stands of Anneli and Feedway (Fig. 2). Estimated root biomass in pure stands differed between locations and varieties, especially in the topsoil. In the 0–20 cm layer, root biomass of Feedway was similar in both locations, whereas root biomass of Anneli in the same layer was more than 2.5 times higher in Taastrup than in Uppsala. Root biomass of Feedway in the 20–40 cm layer in Uppsala was similar to that in the 0–20 cm layer. In Taastrup, however root biomass in the 20–40 cm layer was around 30% lower than in the 0–20 cm layer. Similarly, root biomass of Anneli was comparable between the 0–20 cm and 20–40 cm layer in Uppsala, while in Taastrup, root biomass of Anneli was more than 60% lower in the 20–40 than in the 0–20 cm layer. In the deepest layer, i.e., from 40 to 60 cm depth, similar root biomass values were obtained for the two varieties in both locations (Fig. 2).

The differences in root biomass within single layers of the pure stands between varieties and locations also resulted in different total root biomass. Anneli grown in Taastrup had between 40 and 50% higher total estimated root biomass than Anneli grown in Uppsala or Feedway grown in either location (Table 2).

**Table 2:** Total estimated root biomass from the surface to 60 cm depth in feedway pure stands, Anneli pure stands, and variety mixture in the two locations. To obtain the total root biomass per m^2^, root biomass (mg dry root g^− 1^ dry soil) in each layer was multiplied by the corresponding bulk density and the layer thickness

Location	Variety	Root biomass [g m^− 2^]
Taastrup	Anneli	1006
	Feedway	507
	Mixed	1248
Uppsala	Anneli	621
	Feedway	724
	Mixed	658

### Relative contribution of feedway and Anneli to root biomass in the variety mixture

**Fig. 3:**
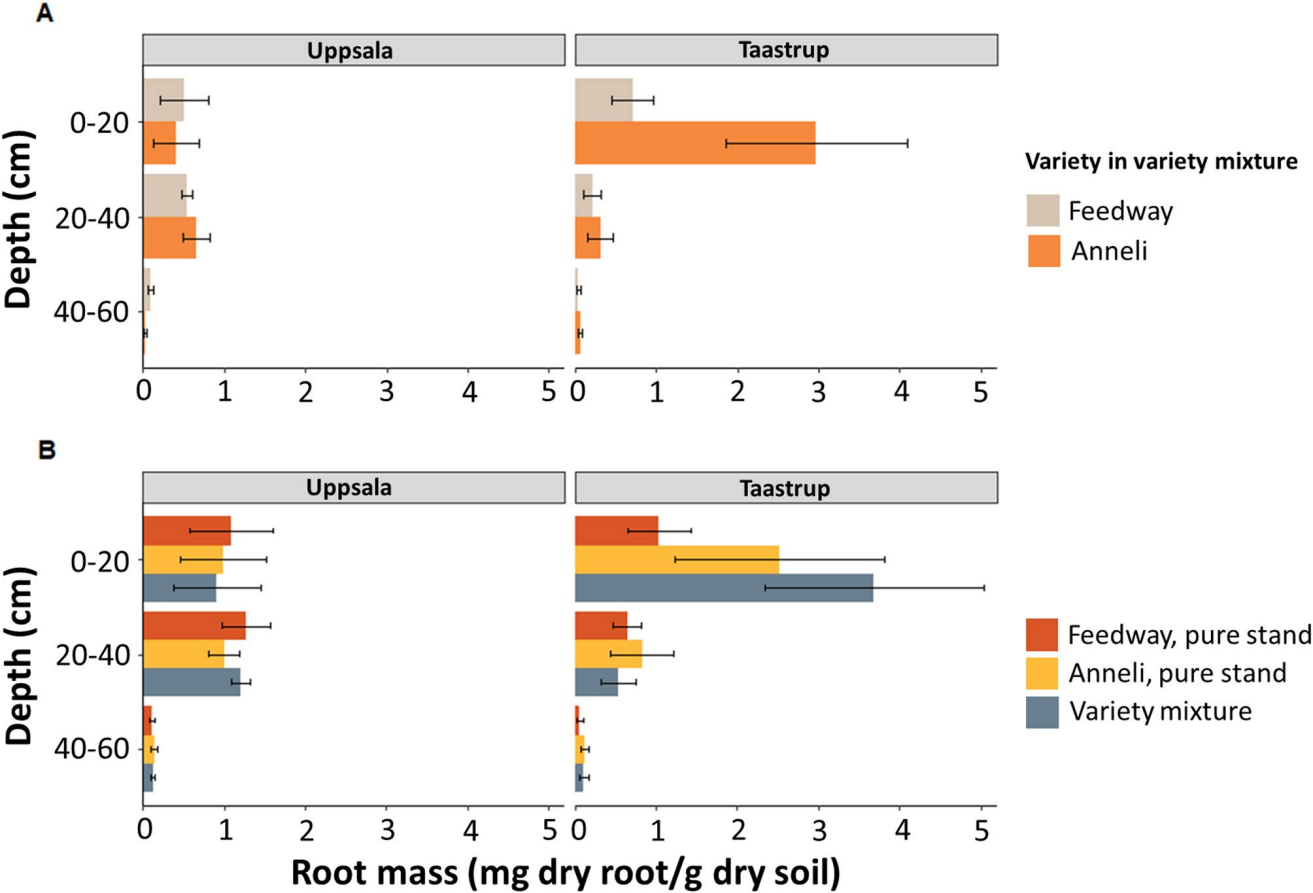
Contribution of Feedway and Anneli to total root biomass in variety mixtures. (A) Root biomass of Feedway and Anneli grown in variety mixtures across different soil layers. (B) Total root biomass in variety mixtures compared to total root biomass in pure stands across different soil layers. The bars represent mean values in root biomass and error bars represent standard errors (*n* = 4)

The variety specificity of the primers allowed us to evaluate the relative contribution of Feedway and Anneli roots to the total root biomass in the variety mixtures (Fig. 3). In Uppsala, Feedway and Anneli had a similar contribution to total root biomass in the 0–20 cm and the 20–40 cm layer (Fig. 3A). Total root biomass in the uppermost 20 cm of the variety mixture (0.9 mg root/g soil ± 0.5 SE) was similar to the root biomass in Feedway pure stands (1.1 mg root/g soil ± 0.5 SE) and Anneli pure stands (1.0 mg root/g soil ± 0.5 SE; Fig. 3B). Similar root biomass in the variety mixture and pure stands was also obtained in the 20–40 cm layer (Fig. 3B). In Taastrup, the relative contribution of Anneli roots to the total root biomass in the top 20 cm in the variety mixture was more than four times higher than that of Feedway (Fig. 3A). Moreover, the total root biomass in the uppermost 20 cm of the variety mixture was around 3.5 times higher than that in Feedway pure stands and around 1.5 times higher than that in Anneli pure stands (Fig. 3B). Root biomass in the variety mixture in Taastrup was considerably higher in the 0–20 cm layer than in the deeper layers (Fig. 3A), which corresponds to the results obtained from Anneli pure stands in Taastrup (Fig. 2). In the 20–40 cm and 40–60 cm layers, total root biomass was similar in the variety mixture and the pure stands (Fig. 3B). As for pure stands, the total root biomass (0–60 cm) in the variety mixture differed between locations, being almost twice as high in Taastrup compared to Uppsala (Table 2).

### Rooting depth

**Fig. 4:**
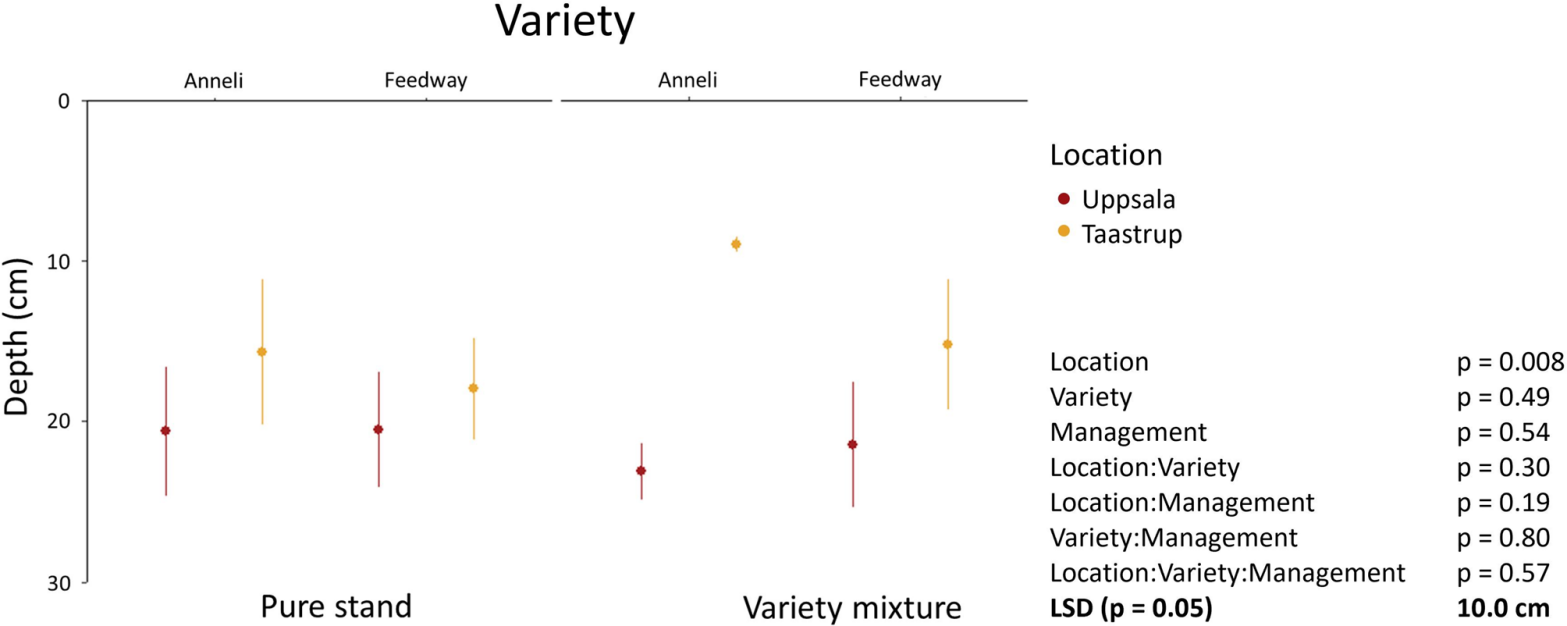
The depths above which 75% of total root mass (D_75_) of Feedway and Anneli grown in Uppsala and Taastrup as pure stands or as variety mixtures occurred. P-values are derived from analysis of variance including the location, the variety, the management (pure stand vs. variety mixture) and their interactions as fixed factors. Least significant differences (LSD) at *p* = 0.05 were derived from analysis of variance. Error bars represent standard error (*n* = 4)

We used the depth above which 75% of total root biomass occurred (D_75_) as a surrogate metric for relative rooting depth. We found a significant effect of the location (*p* = 0.008) on D_75_, whereas no significant effects were observed for the variety, management (i.e. pure stand vs. variety mixture) or the different interactions (*p* ≥ 0.19). The most pronounced difference in D_75_ occurred for Anneli grown in variety mixtures (Uppsala: 23.1 cm ± 1.7 SE; Taastrup: 8.9 cm ± 0.5 SE), followed by Feedway grown in variety mixtures (Fig. 4).

## Discussion

In the current study, we developed a method to quantify root biomass based on the extraction of root DNA from soil that allows estimation of root biomass without washing of roots. Using variety-specific SNP markers, we were able to distinguish individual barley varieties grown together in the same plot as variety mixtures. Furthermore, field experiments in two different locations with contrasting soil types revealed site- and variety-specific root distribution patterns in pure stands and variety mixtures.

### Capability of SNP markers to quantify variety-specific root biomass

We extracted root DNA directly from soil in order to quantify root biomass under field conditions. Other than previous studies that used species-specific ITS sequences to detect root DNA [19–21], we used variety-specific SNP markers to detect and quantify barley roots in soil. This reliance on SNP markers enabled us to design specific primers to estimate the root biomass of individual varieties grown together in the same plots as a mixture. The diploidy of the barley genome facilitated the use of SNP markers to distinguish root DNA between the two varieties. To adapt our method to polyploid crops such as wheat or sugar cane, larger SNP chips offering greater reading depth than the 15 K chip used in the current study might be required [34]. Similarly, larger SNP chips will enable to distinguish genotypes with much lower SNP divergence, which could be especially relevant when mixing a larger number of different accessions from the same population.

As the Anneli- and Feedway-specific primers resulted in negligible or no amplification from the non-targeted varieties (Supplemental Fig. S2), the single nucleotide 3´- end mismatch efficiently prevented amplification. The different regressions slopes of the biological standards (Fig. 1) suggested that DNA recovery differed between varieties and soils, which highlights the need to construct variety- and soil-specific standards. Furthermore, we found that both variety-specific primers enabled root biomass estimation of the target variety across different soil layers until 60 cm depth (Figs. 2 and 3). Hence, using variety-specific DNA markers and the corresponding primers allowed us to detect and quantify roots of individual varieties grown together in mixtures from the surface down to the subsoil. Such differentiation between varieties down to 60 cm is - if at all - hardly possible with established methods relying on root crown excavation and root washing, root quantification along trenches, or (mini-)rhizotrons [17, 18].

### Potential overestimation of root biomass by PCR

Total root biomass in the top 60 cm of the soil estimated from marker copy numbers was between 500 and 1250 g m^− 2^ (Table 2), which is considerably higher than reported in previous studies. In barley, root biomass from 400 to 750 g m^− 2^ in the top 40 cm at flowering have been found [35]. At maturity, barley root biomass ranged from 173 to 350 g m^− 2^ in the top 60 cm [36] and from 348 to 477 g m^− 2^ in the top 30 cm [37]. Hence, we obtained 0.25 to 2.5 times higher root biomass values than previous studies that quantified root biomass by washing roots from soil cores (Supplemental Table S1). Differences in climate conditions, soil type, agricultural management, and the grown barley varieties make direct comparisons between these and our study difficult. Nevertheless, it is evident that our SNP markers-based estimations of root biomass estimations were considerably higher than root biomass reported in studies that washed roots from soil cores. This potential overestimation must be considered when establishing linkages between PCR-derived root biomass estimations and root functions such as soil exploration and soil organic matter input. In our opinion, the data collected in the current study does not allow for a conclusive statement explaining this overestimation. Nevertheless, we identified two potential explanations underpinning the comparatively high root biomass obtained with our method compared to studies that washed barely roots from soil cores [35–37].

First, the difference in tissue age between the roots used to make the biological standards, which were taken from four-week-old plants, and the sampled roots from the field may have resulted in an overestimation of root biomass. The DNA concentration in roots, i.e. the amount of DNA per g of dry root biomass, changes with root age due to alterations of in the biochemical composition of roots, such as a the degree of lignification [20, 38]. Similarly, root DNA concentration is likely to be affected by root structural changes occurring with progressing plant development, such as aerenchyma formation or cortical senescence. Moreover, despite careful washing, young fine roots with a high DNA concentration might have been lost during cleaning of the roots used for the biological standards. We therefore suggest that for future studies, the biological standard used to convert copy numbers of DNA markers to root biomass should be made with roots of similar age and with a similar trait distribution as those sampled in the field.

The second reason causing a potential overestimation of root biomass is that in soil, root DNA is not only found in root biomass. Even though our method does not explicitly separate dead from living roots, as is sometimes done during root washing [36, 39], we likely quantified mainly living roots as the DNA of dead roots rapidly decays in soil [20]. However, both field experiments were located at relatively high latitudes (55° to 60°N) where DNA decay is slower, especially in the colder subsoil, it cannot be excluded that dead root tissues were detected by our method and contributed to the discrepancy between the root biomass data obtained here and elsewhere [35, 36, 39, 40]. Sloughed-off root cap cells constitute a major part of rhizodeposition [41] and are (if the DNA was not degraded) detected with our method but not by root washing, which could have contributed to an overestimation of root biomass. Hence, while our method likely resulted in an overestimation of root biomass, it might deliver more accurate estimates of the root tissue-derived soil carbon inputs than root washing.

### DNA-based root quantification offers new insights into the underground functioning of mixed variety systems

The method presented here has the potential to improve our understanding of belowground processes underpinning the functioning of cropping systems, and variety mixtures in particular. Using variety-specific primers, we could distinguish between roots of the two varieties in the top- and the subsoil of mixed-variety plots (Fig. 3). This capability is essential to elucidate the potential of variety mixtures for belowground niche complementarity, as found in polycultures (Zhang et al., 2014). In addition to the varieties that are included in mixtures, root distribution patterns underlying potential niche complementarity also depend on the local (soil) environment. We observed a general effect of the location on rooting depth, with root systems being shallower in Taastrup than in Uppsala (Fig. 4). The fields at the two locations differed in soil texture, which is known to affect root distribution [42–44]. Furthermore, we found that the contribution of the two varieties to total root biomass in the mixed variety plots differed considerably between the two locations. In Uppsala, the contributions of Feedway and Anneli to total root biomass were comparable across the three depths. In Taastrup, however, Anneli developed much more roots than Feedway, especially in the uppermost 20 cm (Fig. 3). Since the current study only includes data from one growing season, these results should be taken with caution, but they might indicate that in Taastrup, Anneli outcompeted Feedway for topsoil resources.

Our results demonstrate that DNA markers are useful to obtain information about variety- and location-specific root distribution patterns along soil profiles in variety mixtures (Figs. 3 and 4). Such insights cannot be obtained with root crown excavation and root washing or through the use of trenches and (mini-)rhizotrons [17, 18]. We therefore propose that a combination of our method with measurements of plant performance will enable novel insights into the linkages between root distribution, plant-plant interactions (e.g. competition), water and nutrient uptake patterns, and crop productivity in variety mixtures.

## Conclusions

In this study, we present a method to quantify root biomass of different barley varieties that were grown together in the same plot based on the abundance of variety-specific SNP markers in soil. The markers used here were highly variety-specific, which enabled accurate detection of roots of the two varieties. We found distinctly different vertical root distribution patterns of the two varieties grown as pure stands and as mixtures between two locations, highlighting the importance of environmental effects for root distribution under field conditions. We conclude that our method is a valuable tool for future research on the functioning of mixed variety systems, and to evaluate the potential of such systems to improve the sustainability of crop production. The necessary next steps will be to test the adaptability of the presented method across larger numbers of environments and multiple growing seasons and to evaluate its suitability in other crop species and mixtures including a greater number of varieties.

## Supplementary Information

Below is the link to the electronic supplementary material.

Supplementary Material 1

## Data Availability

The datasets used and/or analysed during the current study are available from the corresponding author on reasonable request.
