# Supplementary material for: Quantification of root biomass in barley variety mixtures using variety-specific genetic markers
Source: Plant Methods. 2025 Nov 12;21:147. doi: 10.1186/s13007-025-01464-8 (PMC12613679; doi:10.1186/s13007-025-01464-8)
Supplement: Supplementary file 1 — Supplementary Material 1 [file 13007_2025_1464_MOESM1_ESM.docx]

***Supplemental information to***

**Quantification of root biomass in barley variety
mixtures using variety-specific genetic markers**

Mitsuaki Suizu^1,2*^, Björn D. Lindahl^1^, Carsten W. Müller^3,4^, Thomas Keller^1,5^, Tino Colombi^1,6,$^

^1^ Department of Soil and Environment, Swedish University of Agricultural Sciences (SLU), Box 7014, 750 07, Uppsala, Sweden

^2^ Department of Local Produce and Food Sciences, Faculty of Life and Environmental Sciences, University of Yamanashi, 400-8510, 4-4-37 Takeda, Kofu, Yamanashi, Japan

^3^ Department of Geosciences and Natural Resource Management, University of Copenhagen, Øster Voldgade 10, 1350 Copenhagen, Denmark

^4^ Chair of Soil Science, Institute of Ecology, Technische Universität Berlin, 10587 Berlin, Germany

^5^ Department of Agroecology and Environment, Agroscope, Reckenholzstrasse 191, CH-8046, Zürich, Switzerland

^6^ School of Biosciences, University of Nottingham, Sutton Bonington, LE12 5RD, UK

^*^ Corresponding author; email: m.suizu@yamanashi.ac.jp

^$^ Corresponding author; email: tino.colombi@nottingham.ac.uk

Supplemental Table 1: Comparison of root biomass of field grown barley obtained in the current and previous studies. Root biomass in the current study was estimated from root DNA extracted from soil cores; root biomass in cited studies was obtained by washing roots from soil cores.

| **Reference** | **Developmental stage** | **Depth  [cm]** | **Root biomass  [g m^-2^]** |
| --- | --- | --- | --- |
| Current study | Flowering | 0-60 | 507-1248 |
| [1] | Flowering | 0-40 | 400-750 |
| [2] | Maturity | 0-60 | 173-350 |
| [3] | Maturity | 0-30 | 348-477 |

**Cited references**

1. Czyż EA. Effects of traffic on soil aeration, bulk density and growth of spring barley. Soil Tillage Res. 2004;79:153–66.
2. Ahmadi SH, Sepaskhah AR, Zarei M. Specific root length, soil water status, and grain yields of irrigated and rainfed winter barley in the raised bed and flat planting systems. Agric Water Manag. 2018;210:304–15.
3. Bolinder MA, Angers DA, Dubuc JP. Estimating shoot to root ratios and annual carbon inputs in soils for cereal crops. Agric Ecosyst Environ. 1997;63:61–6.

**A**

1 3´-GGACCTTGGC TAGATGGGTC CTTGACGTGG GACTACAGAG GAAACAAACA CGTTTCCTCC-5´

1 5´-CCTGGAACCG ATCTACCC**A**G GAACTGCACC CTGATGTCTC CTTTGTTTGT GCAAAGGAGG-3´

61 3´-GAAGTGAACG TATGTATGTA CTATGTATGT CGAAGGTACA GGGATCC-5´

61 5´-CTTCACTTGC ATACATACAT GATACATACA GCTTCCATGT CCCTAGG-3´

**B**

1 3´-CTAGTGAAGT ACCAAAGGGG AGTGCCGTAC TGACTGGCGA GGTCGAACCT GAAGGTTCGC-5´

1 5´-GATCACTTCA TGGTTTCCCC T**C**ACGGCATG ACTGACCGCT CCAGCTTGGA CTTCCAAGCG-3´

61 3´-CACCTGCCGC AAGGAAGAGG ACCTCATGGT GGTCCGTCCA CTTCTGCTCG TGTATTATAC-5´

61 5´-GTGGACGGCG TTCCTTCTCC TGGAGTACCA CCAGGCAGGT GAAGACGAGC ACATAATATG-3´

121 3´-GAACAAGGGC ATCACTACAG-5´

121 5´-CTTGTTCCCG TAGTGATGTC-3´

Supplemental Fig. S1. (A) Genetic sequences of Feedway SNP marker in BOPA1_10669-188, and (B) Anneli SNP marker in BOPA1_1286-990. The underlines indicate primer binding sites. The bold capital letters indicate SNP sites. The adenine at the nineteenth position in the Feedway SNP marker shown in the panel A is replaced with guanine in Anneli genome, and the cytosine at the twenty-second position in the Anneli SNP marker shown in the panel B is replaced with guanine in the Feedway genome.


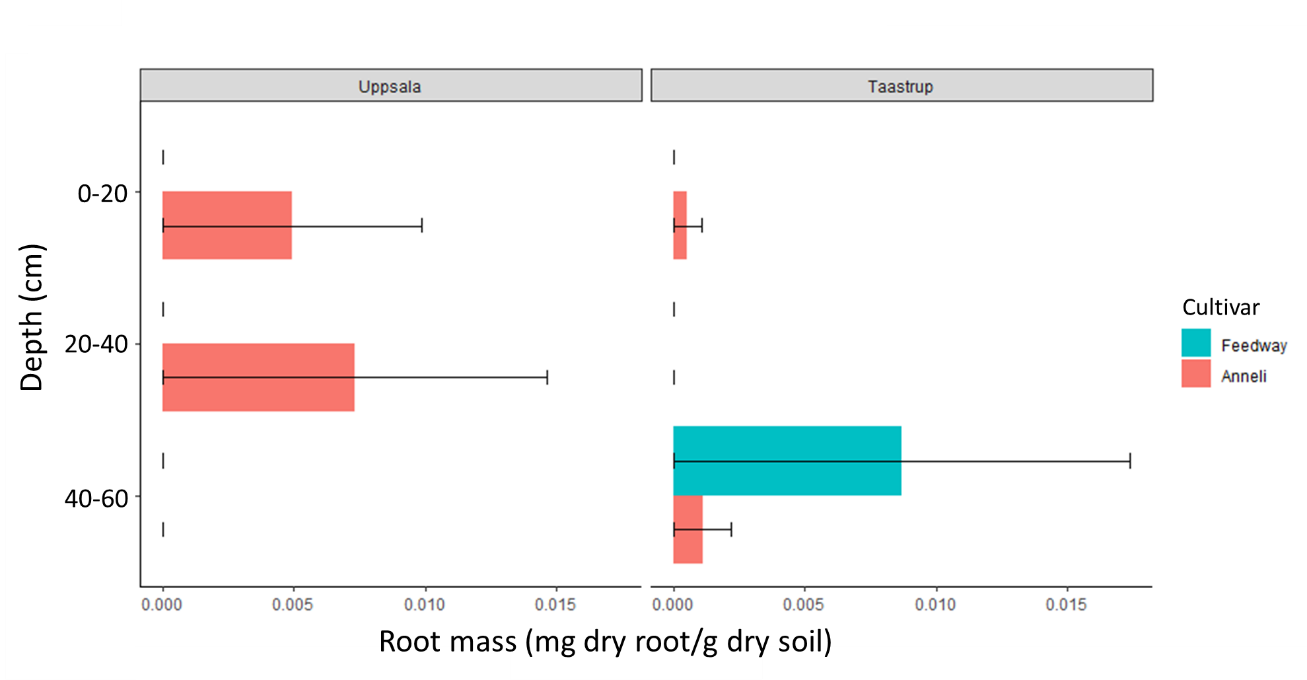


Supplemental Fig. S2. Detection of Feedway and Anneli grown in pure stand using the alternate variety-specific primers (i.e. Feedway SNP marker in Anneli plots and Anneli SNP marker in Feedway plots). The bars represent the mean values of Feedway and Anneli root mass across the soil layers in Uppsala and in Taastrup based on the amplifications with the opponent primers. Error bars represent standard errors (n = 4). Note that the scale of the x-axis is more than 300 times smaller than in Fig. 3.
